# Prevalence and determinants of risky sexual practice in Ethiopia: Systematic review and Meta-analysis

**DOI:** 10.1186/s12978-017-0376-4

**Published:** 2017-09-06

**Authors:** Achenef Asmamaw Muche, Getachew Mullu Kassa, Abadi Kidanemariam Berhe, Gedefaw Abeje Fekadu

**Affiliations:** 1 Department of Epidemiology and Biostatistics, Institute of Public Health, University of Gondar, Gondar, Ethiopia; 2 College of Health Sciences, Debre Markos University, Debre Markos, Ethiopia; 3 College of Medicine and Health Sciences, Adigrat University, Adigrat, Tigray Ethiopia; 4 School of Public Health, College of Medicine and Health Sciences, Bahir Dar University, Bahir Dar, Ethiopia

**Keywords:** Risky sexual practices, Peer pressure, Substance use, Pornography, Gender, Ethiopia, Systemic review, Meta-analysis

## Abstract

**Background:**

Risky sexual practice is a major public health problem in Ethiopia. There are various studies on the prevalence and determinants of risky sexual practice in different regions of the country but there is no study which shows the national estimate of risky sexual practices in Ethiopia. Therefore, this review was conducted to estimate the national pooled prevalence of risky sexual practice and its risk factors in Ethiopia.

**Methods:**

The Preferred Reporting Items for Systematic Reviews and Meta-Analyses guideline was followed to review published and unpublished studies in Ethiopia. The databases used were; PubMed, Google Scholar, CINAHL and African Journals Online. Search terms were; risky sexual behavior, risky sexual practice, unprotected sex, multiple sexual partner, early sexual initiation, and/or Ethiopia. Joanna Briggs Institute Meta-Analysis of Statistics Assessment and Review Instrument was used for critical appraisal. The meta-analysis was conducted using Review Manager software. Descriptive information of studies was presented in narrative form and quantitative results were presented in forest plots. The Cochran Q test and *I* ^2^ test statistics were used to test heterogeneity across studies. The pooled estimate prevalence and the odd ratios with 95% confidence intervals were computed by a random effect model.

**Results:**

A total of 31 studies with 43,695 participants were included in the meta-analysis. The pooled prevalence of risky sexual practice was 42.80% (95% CI: 35.64%, 49.96%). Being male (OR: 1.69; 95% CI: 1.21, 2.37), substance use (OR: 3.42; 95% CI: 1.41, 8.31), peer pressure (OR: 3.41; 95% CI: 1.69, 6.87) and watching pornography **(**OR: 3.6; 95% CI: 2.21, 5.86) were factors associated with an increase in risky sexual practices.

**Conclusions:**

The prevalence of risky sexual practices is high in Ethiopia. Being male, substance use, peer pressure and viewing pornographic materials were found to be associated with risky sexual practices. Therefore, life skills training is recommended to reduce peer pressure among individuals. Interventions should be designed to reduce substance use and viewing pornography.

## Plain English summary

Risky sexual practices increase the likelihood of sexually transmitted infections, unintended pregnancies and unsafe abortion. Ethiopia is one of the sub-Saharan countries with a high burden of sexual and reproductive health problems, risky sexual practices increase this burden. The prevalence of risky sexual practices varies from place to place in Ethiopia. Poor knowledge of sexual and reproductive health services, peer pressure, substance use and poverty are factors associated with risky sexual practices.

There were several studies on prevalence and determinants of risky sexual practices in Ethiopia; however, none show the magnitude and determinants at the national level. Therefore, this review was conducted to identify the prevalence and determinants of risky sexual practices in Ethiopia. Thirty-one articles with 43,695 participants were included. The review identified risky sexual practice as a common public health problem in Ethiopia. About 43% of the participants reported being involved in risky sexual practice. In this review, males were more likely to engage in risky sexual practices than females. Substances users were also more likely to engage in risky sexual practices. Individuals who reported the influence of others to reach decision were more likely to engage in risky sexual practices. Similarly, people who reported viewing pornography were more likely to engage in risky sexual practices. Therefore, governmental and non-governmental organizations should work to address the identified determinant factors to reduce the high burden of risky sexual practice.

## Background

Risky sexual behavior is defined as any sexual activity that increases the risk of contracting sexually transmitted infections (STI) and unintended pregnancies. It includes having sex with multiple sexual partners, not using or inconsistent condom use, sex under the influence of substances and initiation of sex before the age of 18 years. An individual is considered as engaging in risky sexual practice if she/he experienced one or more of the above behaviors [1].

Risky sexual practices increase the risk of HIV/AIDS, unintended pregnancy, unsafe abortion and psychosocial problems [2]. These problems can be aggravated by low income, job insecurity, lack of awareness about sexual and reproductive health issues and harmful traditional practices [3].

Studies showed that poor knowledge of sexual and reproductive health services, peer pressure, substance use, low economic conditions, and poor supervision by parents are factors which can lead to risky sexual practices [4, 5]. Additionally, globalization, curiosity, and enthusiasm of persons to try new things, especially young people, increase risky sexual practices [5].

The Ethiopian government has developed and implemented various strategies to promote sexual and reproductive health [6]. Despite these interventions, different studies in the country showed a high prevalence of risky sexual practices. This level ranges from 13% in Humera to 27% in Addis Ababa [7–9]. These studies showed inconsistent findings of the prevalence of risky sexual practices. Therefore, this study was conducted to summarize the current evidence on the prevalence and determinants of risky sexual practices in Ethiopia.

## Methods

### Study design and search strategy

A systemic review and meta-analysis was conducted using published and unpublished research on the prevalence of risky sexual practices and its associated factors in Ethiopia. All relevant published studies in the following major databases; PubMed, Google scholar, CINAHL, and African Journals Online were included in the review. All published and unpublished articles up to December 30, 2016 were included in the review. The reference lists of identified studies were also reviewed to retrieve additional articles. Unpublished studies were retrieved from the official website of Addis Ababa University electronic database [10]. The following search terms were used: risky sexual practice, multiple sexual partners, commercial sex worker, early sexual initiation, premarital sex, inconsistent use of condom, unprotected sexual activity, sex under the influence of substances, sex with commercial sex workers and Ethiopia separately and/or in combination. The Preferred Reporting Items for Systematic Reviews and Meta-Analyses (PRISMA) guideline was followed during the systematic review [11].

### Study selection and eligibility criteria

The following criteria were used to determine the eligibility of studies.

#### Inclusion criteria:

- Participants: this review included studies that were conducted on risky sexual practices and its determinants in Ethiopia. The participants were people of all ages regardless of their sex and occupation.
- Setting: Studies conducted at community or institutional level in Ethiopia.
- Outcome: The prevalence of risky sexual practices using the communicable disease control (CDC) definition [1].
- Type of study: All study types were included in the review.
- Publication types: journal articles, master’s thesis and dissertations
- Only studies in English were included in the review.

#### Exclusion criteria:

- Studies with the methodological problems and review articles were excluded from the review.
- Retrieved articles were assessed for inclusion using their title, abstract and then a full-text review of articles for quality was done before inclusion in the final review.

### Data extraction

The data extraction was done by four researchers using a data extraction tool. This tool included information on the title, author, year of survey and publication, study design, sample size, data collection procedure, study participants, study area, response rate, sampling method and the definition used for risky sexual practices.

### Quality assessment and data collection

The Joanna Briggs Institute Meta-Analysis of Statistics Assessment and Review Instrument (JBI-MAStARI) was used for critical appraisal [12]. This tool contains a separate appraisal checklist for each type of the study design. Two reviewers independently assessed articles prior to inclusion in the final review using this instrument. Any disagreement which arose between the reviewers was resolved through discussion, and by involving a third reviewer. Studies with quality assessment score of 50% and above and studies having a response rate of 80% and above were included in the final review.

### Publication bias and heterogeneity

Publication bias and heterogeneity were assessed. To check the publication bias, a funnel plot was used. The distribution of studies and a *p*-value <0.05 were used to declare publication bias. The heterogeneity of studies was checked using Q test and *I* ^*2*^ test statistics. *I* ^*2*^ test statistics results of 25%, 50%, and 75% were declared as low, moderate and high heterogeneity respectively. For the test result, which indicates the presence of heterogeneity, a random effects model was used for analysis.

### Statistical methods and analysis

The meta-analysis was conducted using Review Manager (RevMan) software version 5.3. Forest plots were used to present the combined estimate with 95% confidence intervals (CI). For studies which did not present a standard error (SE), it was calculated using the formula; SE = √p x (1-p)/n in Microsoft excel. The calculated standard error and prevalence rate of each study was then entered into RevMan software to calculate the overall prevalence and its 95% CI. Subgroup analyses were conducted based on the gender, substance use, peer pressure and viewing pornography on the risky sexual practices of study participants.

## Results

### Study selection

**Fig. 1:**
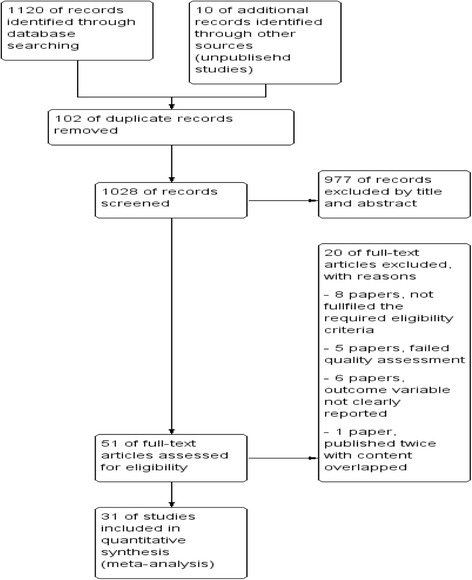
Flow diagram of the studies included in the meta-analysis

The review found a total of 1120 published articles and 10 unpublished reports. From this, 102 duplicate records were removed and 977 records were excluded after screening by title and abstract. A total of 51 full-text articles were screened for eligibility. From this, 20 articles were excluded since they failed to fulfill the eligibility or quality criteria. Finally, 31 studies were included in the analysis **(**Fig. 1 **).**

### Characteristics of included studies

All included articles were cross-sectional studies. Four of the included studies were unpublished [13–16]. The sample size of studies ranged from a minimum of 236, a study conducted among youth in Dilla town, Southern Ethiopia [17] to a maximum of 20,434, a nationwide study [18]. A total of 43,695 participants were included in the review. The studies were conducted from 2001 to 2016 in different regions of the country **(**Table 1 **).**

**Table 1:** Summary characteristics of studies in the meta-analysis to show the prevalence risky sexual behavior in Ethiopia, 2002–2016

S.N	Author, Year	Study design	Year study conducted	Sample size	Study area	Age in years	Response rate	Prevalence (Outcome)	Weakness of the study
1.	(Abebe, Tsion, & Netsanet, 2013)	Institutional based cross sectional study	February 14–28/2012	273	Jimma town	Mean ± SD = 18.6 ± 1.6 Range = 15–24	94.8%	42.1% (Early sexual initiation)	Relatively small sample size
2.	(Abosetugn, Zergaw, Tadesse, & Addisu, 2015)	Community based cross-sectional study design	January, 2012	236	Dilla	Range = 15–24	Not reported	48.3% (Unprotected sex)	Relatively small sample size
3.	(Alamrew, Bedimo, & Azage, 2013)	Institutional based cross sectional study	March 2012	790	Bahir dar city	Mean ± SD = 21.5 ± 2.44	97.6%	45.3%(Having multiple sexual partners)	Not clearly stated
4.	(Bayissa, Mebrahtu, Bayisa, & Mekuanint, 2016)	Institutional based cross sectional study	April 15 to 24, 2015	328	Ambo University	mean age = 22.18 years	87.9%	20.4% (Early sexual initiation)	Not clearly stated
5.	(BELETE, 2016)	Institutional based cross-sectional study	March to April 2016	388	Addis Ababa University	Median age = 21 years	92%	58.2% (having multiple sexual partners)	Might have under reported by Social desirability bias
6.	(Bogale & Seme, 2014)	Institutional based cross sectional study	December; 2011 to January; 2012	826	Shendi town, West GojjamZone	Mean ± SD = 17 ± 1.4 Range = 15–24	97.1%	24.8%(having multiple sexual partners)	Social desirability bias responding cannot be ruled out
7.	(Cherie & Berhane, 2012)	Institutional based cross-sectional study	2011	3543	Addis Ababa,	Majority were in the age group 15–16 year	92.5%	10.6% RSB	Not clearly stated
8.	(Dadi & Teklu, 2014)	Institutional based cross-sectional study design	February to April 2014	422	Humera town	Mean ± SD = 16.89 ± 1.16	100%	13.7% RSB	Might have social desirability bias
9.	(Dagne, 2013)	Institutional based cross-sectional study	December, 2013 toDecember, 2014	700	Debre Markos town	Mean ± SD = 17.1 ± 1.6 years Range = 15–24 years	100%	78.8% (Early sexual initiation)	recall bias
10.	(Debebe, 2015)	Institutional based cross-sectional study	February 1–30, 2014	604	Madawalabu University,	Mean ± SD= 21 ± 1.60 years	95.3%	35.2% (having multiple sexual partners)	Personal and sensitive issues related to sexuality which might have caused underreporting of some behaviors
11.	(Derbie, Assefa, Mekonnen, & Biadglegne, 2016)	Institutional based cross sectional study	February 1- march 30, 2014	343	Debre Tabor University	mean age 21.1 years (SD ±2.3)	87.1%	39.6% (inconsistent condom used)	The study lacks data on the sexual experiences of students before they join the University
12.	(Desale, Argaw, & Yalew, 2016)	Institutional based cross sectional study	June 2014	1332	Lalibela town	Mean ± SD =17.39 ± 1.63 years Range = 15–24 years	97.8%	46.5% RSB	Might have underreporting as it is not free from social desirability bias and a recall bias, especially in estimating age at first sexual experiences.
13.	(Dessie, Berhane, & Worku, 2014)	Community based cross-sectional study	March to July 2012	633	Harar town	Range = 13–18 years	98.75	47.55% RSB	Might have social desirability bias
14.	(Dingeta, Oljira, & Assefa, 2012)	Institutional based cross sectional study	March to April, 2010.	1286	Haramaya University	Mean ± SD = 21.4 ± 1.3	98.9%	28% (Early sexual initiation)	Not clearly stated
15.	(Fite & Cherie, 2016)	Community based cross sectional study	March to June 2014	407	AddisAbaba	Mean ± SD = 16 ± 1.22 years	96.4%	23.6% (Early sexual initiation)	Social desirability bias
16.	(Fury, 2006)	Institutional based cross-sectional study	Jan. 2006 to Feb. 2006	813	Ambo town	Mean ± SD= 17.26 ± 2.1 SD years Range = 10–30 years	96.4%	56.4% (having multiple sexual partners)	recall bias
17.	(G. M. Kassa et al., 2016)	Institutional based cross-sectional study design	April to May 2014	300	Jigaworeda, West Gojjam	Mean ± SD = 17.75 ± 1.37 years	96.8%	56.3% % (Early sexual initiation)	Might have recall bias
18.	(Girma, Hailu, Ayana, & Ketema, 2015)	Institution based cross sectional study	February to March,2014	636	Addis Ababa	Mean ± SD = 17.86 ± 0.97 Range = 15–24	97.85%	25.3% (Early sexual initiation)	Not clearly stated
19.	(Gizaw, Jara, & Ketema, 2014)	Institutional based cross sectional study	Apri1.7–25, 2014	836	Addis Ababa	Mean = 17.3 Range = 15–24	99.3%.	26.7% RSB	Response may affected by social desirability bias
20.	(Guta & Yeshambel, 2016)	Institutional based cross sectional study	April 15 to 24, 2015	328	Ambo University	Mean age = 22.18 years	87.9%	20.4%(Early sexual initiation)	Not clearly stated
21.	(Henok, Kassa, Lenda, Nibret, & Lamaro, 2015)	Institutional based cross-sectional study design	June to July 2014.	284	Mizan-Tepi University	18–23 years (80.3%)	100%	44.5% (having multiple sexual partners)	Relatively small sample size
22.	(Kebede et al., 2005)	DHS based survey	December 2001 and May 2002	20,434	Ethiopia	Range = 15–24 years	91.2%	20% (unprotected sex)	Not clearly stated
23.	(Mulu, Yimer, & Abera, 2014)	Institutional based cross sectional study	December to February2013.	817	Bahir Dar University	mean age = 21 years Range = 18–30 years	97.1%	62% (Unprotected sex)	Social desirability bias
24.	(Regassa, Chala, & Adeba, 2016)	Institutional based cross-sectional study	February to March 2014	704	Wollega University	median age of respondents was 21 years	99.86%	62.1% (Early sexual initiation)	Not clearly stated
25.	(Seme & Wirtu, 2016)	Institution based cross sectional study	February andMarch 2006	676	Nekemte	Mean = 16 years	93.6%	57.2% (Early sexual initiation)	Not clearly stated
26.	(T. A. Kassa, Luck, Birru, & Riedel-Heller, 2014)	Cross-sectional survey	June toSeptember 2012	426	Addis Ababa	Mean ± SD = 20.8 ± 2.6 years	100%	75. 1% (Early sexual initiation)	Social desirability bias
27.	(Tadesse & Yakob, 2015)	Community based cross-sectional study	September2011	711	Tiss Abay	Mean ± SD = 21.54 ± 3.84 years Range = 15–29 years	95.9%	70.3% RSB	Not clearly stated
28.	(Teferra, Erena, & Kebede, 2015)	Institutional based cross sectional study	March 1 -may30 2013	302	MadawalabuUniversity	Range = 15–34	93.2%	33.6%(having multiple sexual partners)	Not clearly stated
29.	(Teshome & Gedif, 2013)	Institutional based cross-sectional study design	November to December 2010	2551	Addis Ababa	Mean ± SD = 16.93 ± 1.35 yearsRange = 14–25 years	92.4%)	52.5% RSB	Not clearly stated
30.	(Tiruneh, Wasie, & Gonzalez, 2015)	Community-based cross-sectional study	July 8–18, 2013	756	Metema District	Median age 22 years and IQR = 20–25	100%	74% RSB	Social desirability responding cannot be ruled out
31.	(Tura, Alemseged, & Dejene, 2012)	Institutional based cross-sectional study	November 2009	1010	Jimma University	Mean ± SD = 17.7 ± 2.7 years	80%	28.3%(having multiple sexual partners)	Social desirability bias might result under reported

*RSB* Risky Sexual Behavior

### Prevalence of risky sexual practice in Ethiopia

The highest prevalence of risky sexual practice was reported in a study among in-school youth in Debre Markos town, Northwest Ethiopia. The study showed that 78.8% initiated sex at an early age (< 18 years) [13]. The lowest prevalence of risky sexual practice was 10.6% among school adolescents in Addis Ababa, Ethiopia [19].

**Fig. 2:**
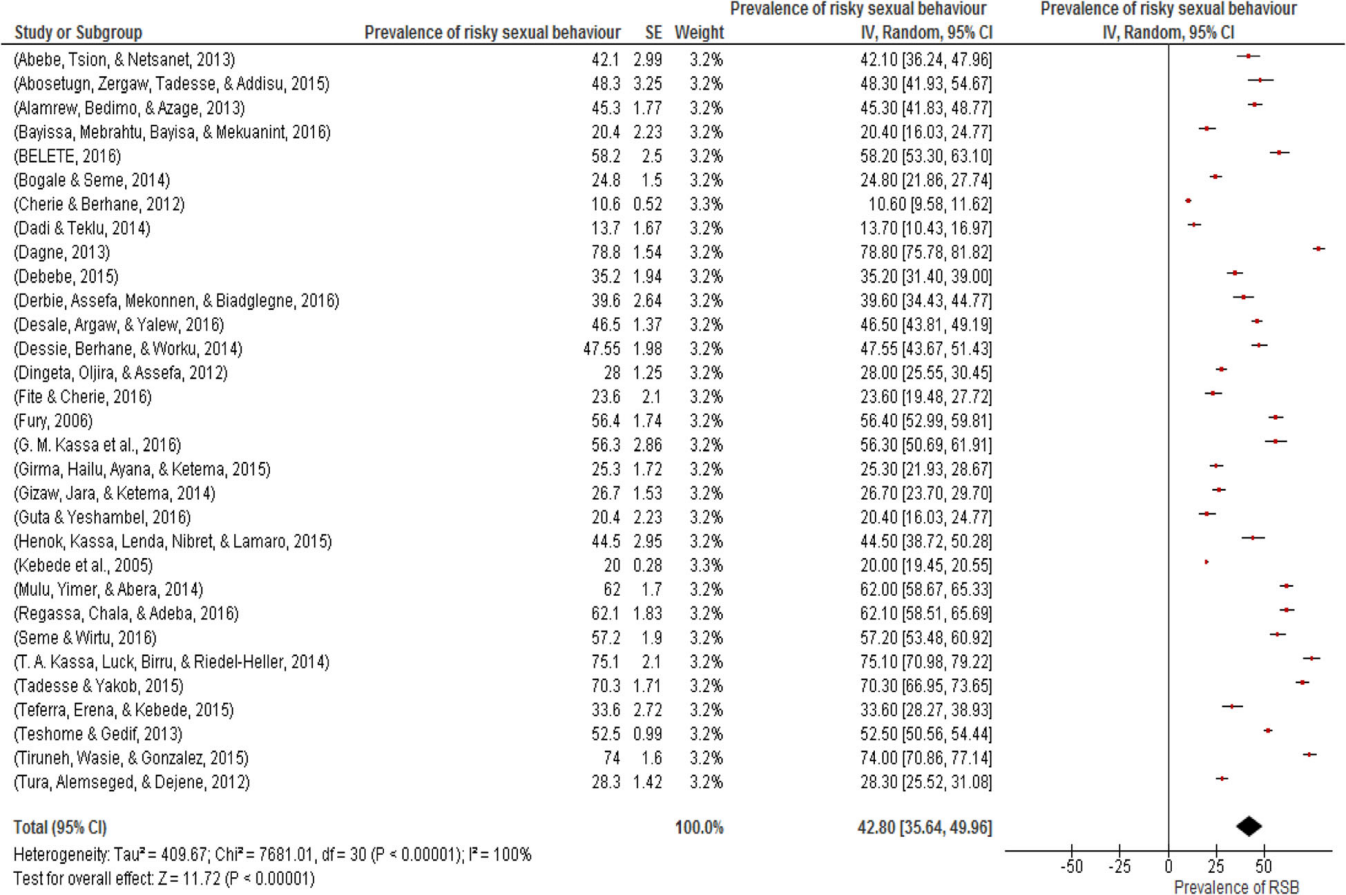
Prevalence of risky sexual practice in Ethiopia, 2002–2016

As presented in Fig. 2, the *I* ^*2*^ test result showed high heterogeneity (I^2=^100%), which is indicative of using random effects model. The overall pooled prevalence of risky sexual practice in Ethiopia was 42.8% (95% CI: 35.64%, 49.96%) **(**Fig. 2 **).**

### Gender disparity and risky sexual practice

**Fig. 3:**
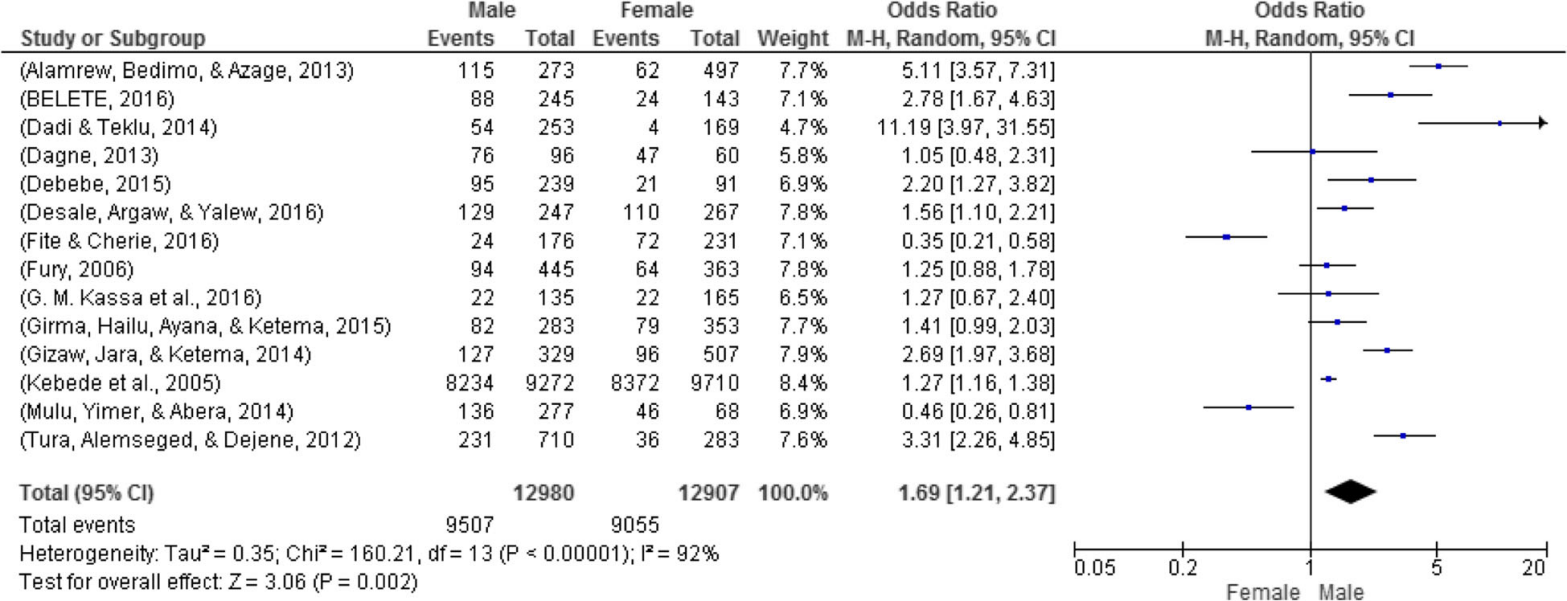
Sex and risky sexual practice in Ethiopia, 2002–2016

A total of 14 articles with 25,887 participants were included to assess the association of gender and risky sexual practices [13–16, 18, 20–28]. The findings showed a higher prevalence of risky sexual practice among males (73.24%) compared to females (70.16%). There was a significant association between gender and risky sexual practices. Males were 1.69 times more likely to engage in risky sexual practices compared to females (OR: 1.69, 95% CI: 1.21, 2.37) **(**Fig. 3 **).**

### Substance use and risky sexual practice

**Fig. 4:**
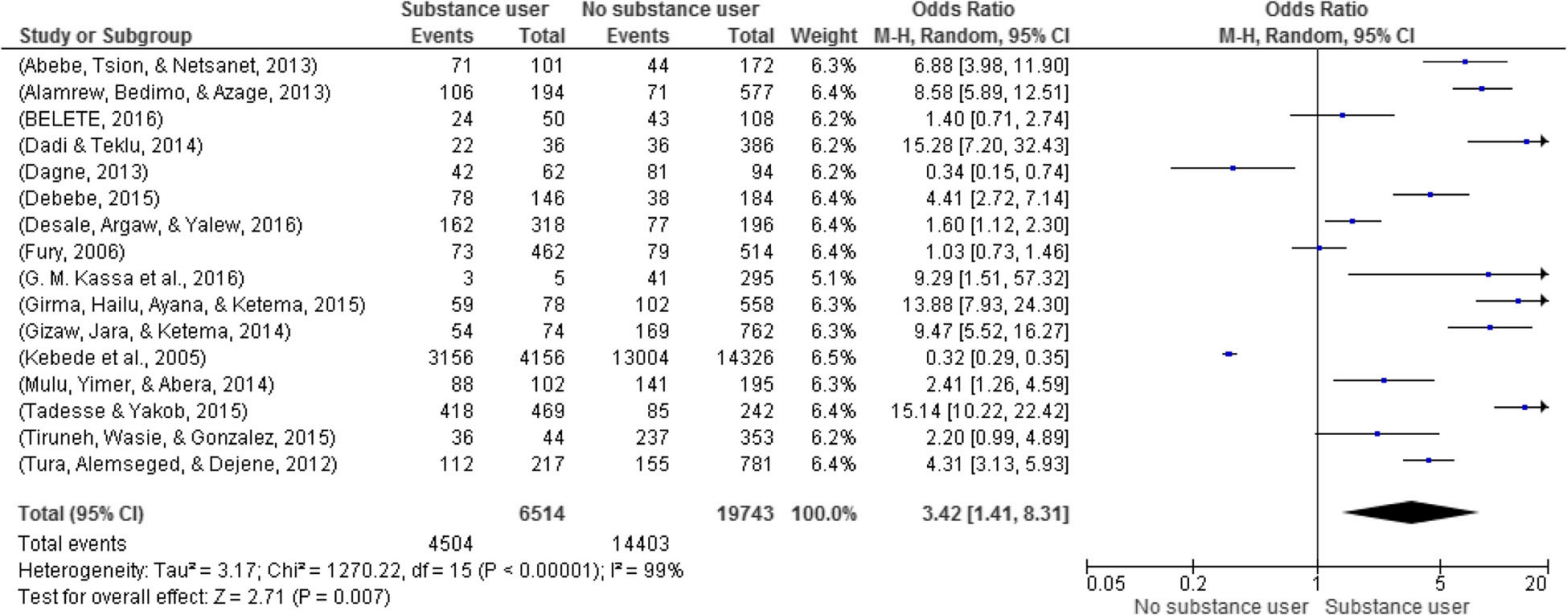
Substance use and risky sexual practice in Ethiopia, 2002–2016

A total of 16 articles with 26,257 participants were included to identify the association between substance use and risky sexual practices [13–16, 18, 20–22, 24–31]. Substance users were 3.4 times more likely engage in risky sexual practices compared to nonusers (OR: 3.42; 95% CI: 1.41, 8.31) **(**Fig. 4 **).**

### Peer pressure and risky sexual practice

**Fig. 5:**
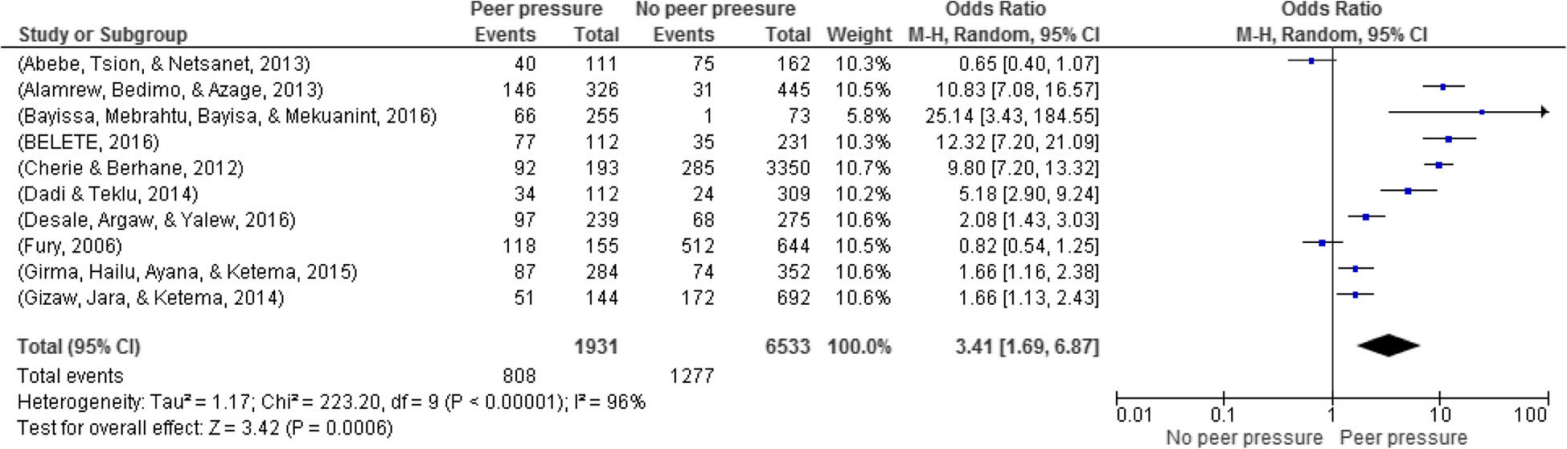
Peer pressure and risky sexual practice in Ethiopia, 2002–2016

Ten articles with a total of 8486 participants were included in the analysis [14, 16, 19–22, 24, 25, 29, 32]. Individuals who reported to be influenced by peers for their risky sexual practice were 3.4 times more likely to engage in risky sexual practices (OR: 3.41: 95% CI: 1.69, 6.87) **(**Fig. 5 **).**

### Viewing pornography and risky sexual practices

**Fig. 6:**
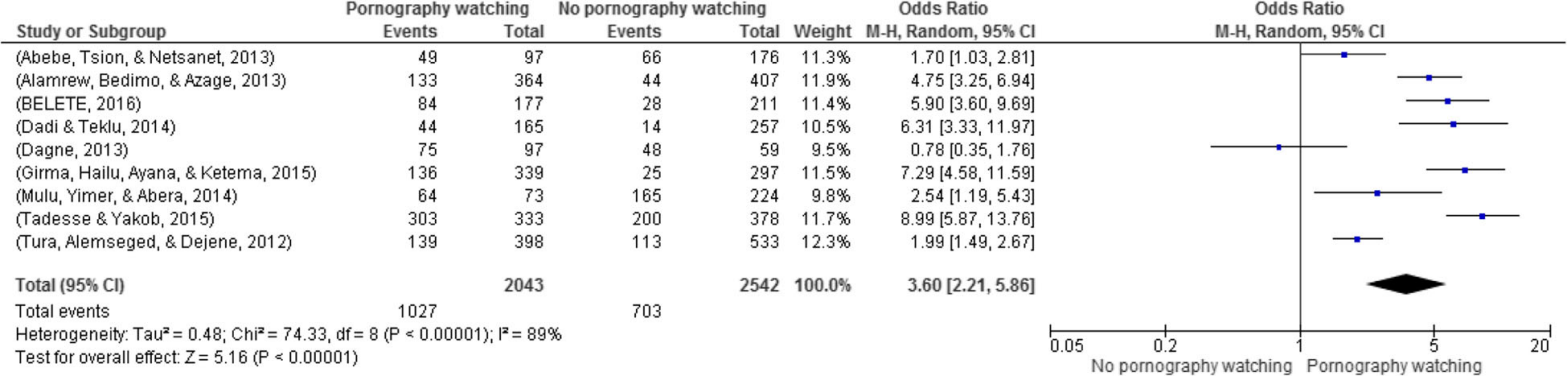
Watching pornography and risky sexual practice in Ethiopia, 2002–2016

Nine articles with a total of 4585 participants were included in this analysis [13, 16, 20, 21, 24, 27–30]. There was a higher prevalence of risky sexual practices among participants who viewed pornography (50.27%) compared to those who did not (27.66%). Risky sexual practices were four times more likely to occur among participants who viewed pornography (OR: 3.6: 95% CI: 2.21, 5.86) **(**Fig. 6 **).**

## Discussion

This review was conducted to identify the prevalence of risky sexual practices and its associated factors in Ethiopia. The overall pooled prevalence of risky sexual practice in Ethiopia was 42.8% (95% CI: 35.64%, 49.96%). This finding was lower than the prevalence of risky sexual behavior (75%) demonstrated among male youth in Latin America [37]. The differences in the findings may reflect differences in participants’ characteristics. This review included both male and female participants, while the other study included only male participants. The other possible explanation could be the difference in the age of participants, this review included both adolescents and adults. On the other hand, the prevalence of risky sexual practice in this review was higher than a meta-analysis of high-risk sexual behavior of women in sub-Saharan countries [38]. The difference may be due to the variation in the study population and sociocultural differences, such as the freedom to talk about sexuality, sex out of marriage, multiple sexual partners and others.

The subgroup analysis of gender and risky sexual practice showed that males were 1.69 times more likely to engage in risky sexual practices compared to females. This finding is in line with a global study on risky sexual behavior using data from 59 countries which showed that risky sexual practices were higher among men than women [33]. The higher prevalence of risky sexual practice among men may be due to a higher proportion involved with multiple sexual partners and higher rate of substance use. The Global School-Based Student Health Survey (GSHS) conducted in Trinidad and Tobago showed that men were more likely to engage in premarital and early sex than women [33]. A large-scale study conducted by Mid-western State University reported that men were more likely to engage in risky sexual practice compared to women [39]. Men are also more likely to have multiple sexual partners compared to women [34, 35].

This review showed that substance users were more likely to engage the risky sexual practice. This finding was similar to studies conducted by WHO and UNAIDS in eight countries [36] and a meta-analysis conducted in Latin America [37]. The reason for this could be because alcohol use affects decision making which may lead to risky sexual practice.

This study identified peer pressure as an important predictor of risky sexual practice. This finding is in line with a study in Ghana and a meta-analysis conducted in sub-Saharan Africa [38, 39]. The reason for this may be that people do not want to be different or rejected, and they may engage in risky behaviors because of the pressure from friends; this may especially be true for youth.

This study also indicated that viewing pornography was associated with risky sexual practices. A population-based study in Sweden indicated that boys who viewed pornography were more likely to engage in risky sexual practices [40]. Individuals who viewed or read pornographic materials frequently were more likely to have multiple sexual partners compared to those who did not [41]. This might be due to the impulsive nature of pornographic materials which lead to erotic sex stimulation or risky sexual practices.

This review used a comprehensive search strategy and multiple database searches of both published and unpublished articles. Random effect model was used to address the issues of potential variability across studies. Included articles were restricted to English language only; this is a limitation of the study as it missed studies published in other languages. All studies included were cross sectional which cannot show a cause and effect relationship between risky sexual practice and the characteristics presented.

## Conclusion

This systematic review and meta-analysis revealed that the prevalence of risky sexual practice was high in Ethiopia. Being male, substance use, peer pressure and viewing pornographic materials were found to be associated with risky sexual practices. Governments and relevant stakeholders should develop effective programs and interventions to address gender-specific communication and life skills to minimize risky sexual practices. Comprehensive multi-level behavioral interventional programs are needed to reduce peer pressure, substance use and watching pornography and formulating an appropriate strategy to monitor pornography and substance use. The authors recommend large-scale studies to identify other variables which could affect risky sexual practices in the country.

## Abbreviations

AIDS: Acquired Immune Deficiency Syndrome
CINAHL: Cumulative Index to Nursing and Allied Health Literature
EDHS: Ethiopian Demographic and Health Survey
HIV: Human Immunodeficiency Virus
JBI-MAStARI: Joanna Briggs Institute Meta-Analysis of Statistics Assessment and Review Instrument
OR: Odds Ratio
PRISMA: Preferred Reporting Items for Systematic Reviews and Meta-Analyses
SD: Standard Deviation
USAID: United States Agency for International Development
WHO: World Health Organization
